# Transgenic *Bacillus thuringiensis* (Bt) Rice Is Safer to Aquatic Ecosystems than Its Non-Transgenic Counterpart

**DOI:** 10.1371/journal.pone.0104270

**Published:** 2014-08-08

**Authors:** Guangsheng Li, Yongmo Wang, Biao Liu, Guoan Zhang

**Affiliations:** 1 Hubei Insect Resources Utilization and Sustainable Pest Management Key Laboratory, College of Plant Science and Technology, Huazhong Agricultural University, Wuhan, Hubei, P.R. CHINA; 2 Nanjing Institute of Environmental Science, Ministry of Environmental Protection of the People’s Republic of China, Nanjing, P.R. CHINA; University of Tennessee, United States of America

## Abstract

Rice lines genetically modified with the crystal toxin genes from *Bacillus thuringiensis* (Bt) have experienced rapid development, with biosafety certificates for two Bt rice lines issued in 2009. There has still been no commercial release of these lines yet due to public concerns about human health and environmental risks. Some studies confirmed that Bt rice was as safe as conventional rice to non-target organisms when pesticides were not applied, however, pesticides are still required in Bt rice to control non-lepidopteran pests. In this study, we assessed the environmental effects of two Bt rice lines expressing either the *cry1Ab/1Ac* or *cry2A* genes, respectively, by using zooplanktons as indicator species under normal field management practices using pesticides when required. In the whole rice growing season, non-Bt rice was sprayed 5 times while Bt rice was sprayed 2 times, which ensured both rice achieved a normal yield. Field investigations showed that rice type (Bt and non-Bt) significantly influenced zooplankton abundance and diversity, which were up to 95% and 80% lower in non-Bt rice fields than Bt rice fields. Laboratory rearing showed that water from non-Bt rice fields was significantly less suitable for the survival and reproduction of *Daphnia magna* and *Paramecium caudatum* in comparison with water from Bt rice fields. Higher pesticide residues were detected in the water from non-Bt than Bt rice fields, accounting for the bad performance of zooplankton in non-Bt field water. Our results demonstrate that Bt rice is safer to aquatic ecosystems than non-Bt rice, and its commercialization will be beneficial for biodiversity restoration in rice-based ecosystems.

## Introduction

Rice (*Oryza sativa* L.) is a staple food for more than three billion people [1], and more than 90% of total rice is produced and consumed in Asia [2]. Over 100 species of insects attack and damage rice, and lepidopteran pests such as rice stem borers are the most serious with yield losses ranging from 5% to 25% [3]. Rice has been successfully modified to express the cry genes derived from the *Bacillus thuringiensis* (Bt) bacterium, and several Bt rice lines with high resistance to lepidopteran pests have been developed over the past 15 years in China [4]. The China Ministry of Agriculture (CMOA) has confirmed that safety certificates were issued in November 2009 for two Bt rice lines, Huahui no. 1 and Bt Shanyou 63 [5], both of which express a fusion *cry1Ab/1Ac* gene. However, the safety certificates did not lead to final commercial release because there were fierce public debates on the health and environmental risks of Bt rice [6]. There is therefore an urgent need to provide the public with scientifically based knowledge of the actual environmental effects of genetically modified rice.

Many field and laboratory experiments have assessed the impact of Bt rice on paddy arthropods, including parasitoids, predators and non-target herbivores. In general, Bt rice has no negative impact (in comparison to non-Bt rice) to the individual fitness, population abundance and community diversity of non-target arthropods [7]–[13]. However, almost all of the field studies have excluded pesticides from both the Bt and non-Bt rice fields. Although Bt rice is effective in reducing pesticide sprays, some pesticide application is still required to control fungal diseases and non-lepidopteran pests if full yield is to be achieved [14], [15]. To accurately assess the environmental impact of Bt rice it is therefore necessary to conduct assessments under normal agricultural practices, including pesticide sprays when required.

Previous environmental assessments of Bt rice have mainly focused on terrestrial arthropods, and have not considered the aquatic ecosystem [16]. In most growth stages of rice, a layer of water is sustained in the rice field. In tropic or subtropical areas, this paddy water layer teems with zooplankton such as rotifers, cladocerans and copepods, most of which are shared by adjacent ponds and lakes, and those organisms constitute an important food source for fish and predatory insects [17]. These organisms also carry out active functions in nutrient cycling in agroecosystems [18]. Further, pesticides readily dissolve in water, and the environmental effects of pesticide use are likely to be most acute in these ecosystems [19].

**Figure 1:**
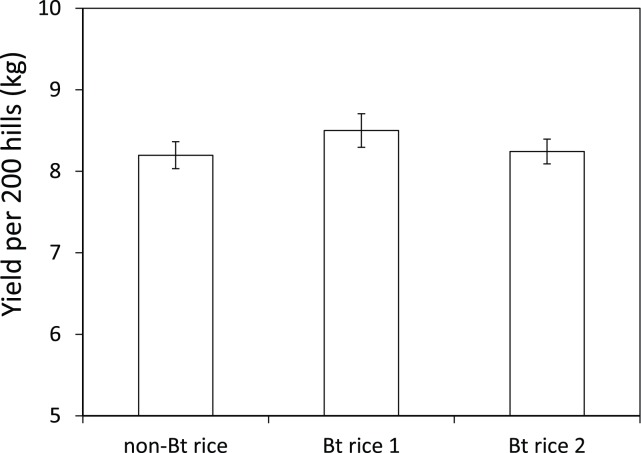
Yield of Bt and non-Bt rice. Non-Bt rice = Minghui 63, Bt rice 1 = Minghui 63 *cry1Ab/1Ac*, Bt rice 2 = Minghui 63 *cry2A*. Non-Bt rice as well as Bt rice were sprayed with pesticides when insect or fungal pests reached the action threshold. Error bars indicate the standard error. Planned comparison of means showed that the yield of non-Bt rice and Bt rice did not differ significantly (d.f. = 6; *t* = 1.303; *p* = 0.240).

In the present study, we evaluated the environmental effects of two Bt rice lines, one of which has been granted a safety certificate by the CMOA, using paddy zooplanktons as indicator species. Bt rice and non-transgenic rice were planted in plots in a complete randomized block design. Experimental fields were inspected weekly for rice pest occurrence and sprayed with pesticides when required to guarantee that non-Bt and Bt rice achieved equal yields (Fig. 1). The diversity and abundance of three types of zooplankton were compared between Bt and non-Bt treatments. Field water was collected to conduct laboratory rearing experiments of water quality using *Daphnia magna* and *Paramecium caudatum*, two species commonly used to evaluate water pollutants [20], [21]. Pesticide residues in field-collected water were also compared between Bt and non-Bt rice fields.

## Materials and Methods

### Ethics Statement

All field studies were carried out at an experimental field which is maintained by Huazhong Agricultural University (HZAU) and located in the campus (latitude 30°34′ N, longitude 114°47′ E). Special permits for the field studies were obtained from Science and Technology Department of HZAU which executes the function of GMO management under the supervision of China National Agricultural GMO Safety Committee (CNAGSC). All materials used during rice planting (pesticides and fertilizer etc.) were registered for legal use, and the products of Bt rice (seed and straw) were disposed according to the regulations formulated by CNAGSC. The field studies did not involve endangered or protected species.

### Bt rice materials and field design

Two Bt rice lines, Bt rice 1 and Bt rice 2, and their common non-transgenic isogenic counterpart, were used as plant materials. Both the Bt rice lines are variety of Minghui 63, an elite indica rice restorer line. Bt Rice 1 was transformed with a synthetic *cry1Ab/1Ac* gene and Bt rice 2 was transformed with the *cry2A* gene [22], [23]. Bt rice 1 is also called Huahui no. 1, which was issued with a safety certificate by the CMOA in 2009 [5]. Field experiments were carried out at an experimental field at the HZAU campus in Wuhan (China) in 2013. The experimental field was constructed from a small hill in 2008, and planted with rice for 3 years without pesticide spray. According to a randomized complete block design (RCBD), the field was divided into 3 blocks, and each block divided into 3 plots which were planted with Bt rice 1, Bt rice 2 and non-Bt rice randomly. Each plot covered an area of 220 m^2^. Solid ridges and drainage channels were built around plots to avoid water flow between the plots. Both Bt and non-Bt rice were sowed in early May and transplanted by hand one month later. Spacing between rows was 25 cm, and hills within rows were 20 cm apart. The field was irrigated with water from a lake nearby. The soil in the experimental field was typically a yellow-brown soil consisting of 25.6% clay, 68.8% silt, 5.6% sand, and 11.2% organic matter. Field management was performed following conventional agronomic practices. Pesticides including avermectins, triazophos, difenoconazole, propiconazole and buprofezin were used when any of the rice pests exceeded action threshold (see Table S1). Action threshold refers to a point at which pest population or environmental conditions indicate that pest control action must be taken to guarantee no economic loss. A windshield was held downwind between plots when spraying.

### Field investigation of zooplanktons

Five litres of water were collected from each plot in the morning before 10 a.m., and were concentrated to 50 ml using plankton net (64 µm mesh size). The concentrated samples were fixed with Lugol’s solution, and stored in amber bottles. Three kinds of zooplanktons, Rotifera, Cladocera and Copepoda, were identified down to the lowest possible taxonomic level, and were counted with the drop count method [24]. Each sample was counted twice and the mean was recorded. The zooplankton investigation was conducted twice, 5 and 12 days after the final pesticide spray on August 1st (see table S1).

### *Daphnia magna* culturing in field-collected water

*Daphnia magna* is highly sensitive to toxic substances in aquatic ecosystem, and has a short generation time [25]. *Daphnia magna* used in the experiments were from a single clonal population cultured in laboratory. Field water was collected from each plot and was filtered by 64 µm plankton net to remove suspended particles and plankton. The filtered water was used as culture medium in the water quality tests. A newly hatched *D. magna* (<24 h old) was transferred to a 100-ml beaker filled with 80 ml filtered field-collected water using a broad-tipped pipette. This was replicated 30 times per plot (9 plots in total). Three millilitres of *Scenedesmus obliquus* solution (4∼5×10^5^ cells/ml), cultivated according to Stein 1973 [26], was added to each beaker as food source for the *D. magna* [27]. Every 3 days, each *D. magna* was transferred to a new beaker with new filtered field-collected water and food source of *S. obliquus*. Each beaker was inspected daily to record survival and presence of newly born *D. magna*, and removal of offspring. All cultures were maintained under static conditions at 23±1°C and a controlled photoperiod at 12 h light and 12 h darkness.

### Field-collected water quality test using *Paramecium caudatum*

*Paramecium* are unicellular organisms belonging to Ciliophora that are commonly found in ponds and lakes. They are often used as bioindicators of aquatic environmental pollutants [28]. Stock cultures of *P. caudatum* were cultured in laboratory according to Ishikawa and Hota’s procedure [29]. Twenty grams of dry rice straw (from a field without pesticide application) was boiled in 2 L tap water for 5 min and the supernatant was used as liquid food of *P. caudatum*. The experimental unit consisted of a 50 ml centrifuge tube with 10 ml culture medium, 3 ml *P. caudatum* stock culture and 37 ml filtered field-collected water. The initial density in the tubes was 54.3 cells ml. Each plot had 5 experimental replicates. The cultures were maintained in a climate chamber at 23±1°C, with a photoperiod of 12 h light and 12 h darkness. The density in each tube was determined every day in the morning using a stereo microscope and counting chamber. This experiment lasted for 8 days.

### Pesticide residue test in field-collected water

Field water samples were collected from each plot on August 11, 2013 (see Table S1 for pesticide application dates). All samples were filtered through a plankton net (64 µm mesh size) and stored into 100 ml amber glass bottles at 4°C. The samples were sent to Technology Service Centre Co., Ltd (http://www.labfaster.com/) the next day, and subjected to liquid chromatography-mass spectrometry (LC-MS/MS) analysis for residues of 5 pesticides. Those pesticides had been used in the experimental field before the sampling date. Each sample was analyzed twice and the average residue was recorded.

### Rice yield test

After rice grain fully ripened in September, 20 rows of rice each containing 10 hills were selected randomly in each plot. Panicles were threshed and rice grain was dried in sunlight and weighed.

### Data analysis

Yield data were analyzed by one-way RCBD ANOVA, and means were compared between non-Bt rice and Bt rice by a planned comparison. Data of zooplankton in field investigation were recorded as each group abundance and total abundance, and those abundances were transformed by ln (x+1) to meet the assumption of homogeneity of variances and then were subjected to one-way RCBD ANOVA. The means of abundance were separated by Fisher’s LSD test. In order to explore the effect of overall rice type on abundance of zooplankton, multivariate analysis of variances (MANOVA) combining each group of zooplankton were also conducted. The survival rates of *D. magna* were arcsine-transformed and subjected to repeated measures ANOVA to estimate rice type and culturing time effect and their interaction; data of total reproduction were square root transformed and analysed by one-way RCBD ANOVA followed by a LSD test. The population growth of *P. caudatum* were also ln (x+1) transformed and subjected to repeated measures ANOVA. All analyses were carried out using general linear model (GLM) in SPSS package (version 16.0, SPSS Inc).

## Results

### Zooplankton abundance and diversity in Bt and non-Bt rice field

Thirty-three species were identified, 17 species in Rotifera, 14 in Cladocera and 2 in Copepoda (see Table S2). In the first investigation, 4, 21 and 18 species were found in non-Bt rice, Bt rice 1 and Bt rice 2 treatment, respectively, and 16, 24 and 19 species in the second investigation (see Table S2). One-way randomized complete block design (RCBD) ANOVAs detected significant rice type effect (d.f. = 2,4; *F*>10.659; *p*<0.035) and non-significant block effect (d.f. = 2,4; *F*<4.515; *p*>0.094) in both investigations on abundance of each zooplankton group (Table 1). The lowest abundance was always found in non-Bt rice plots. In the first investigation, total abundance in Bt rice treatments was around 1,500 individuals L^−1^, which was significantly higher than that in non-Bt rice treatment (below 50 individuals L^−1^) (LSD test, *p*<0.01); the differences between Bt rice 1 and Bt rice 2 were not significant (LSD test, *p* = 0.974). The second investigation found rebound in zooplankton abundance to around 1,600 and 640 individuals L^−1^ in Bt rice and non-Bt rice respectively, but the trend of differences between Bt and non-Bt did not change (Table 1). Multivariate analyses of variance (MANOVA) were used to assess overall rice type effect on abundance of 3 groups of zooplankton, and significant effects were detected in the first investigation (d.f. = 6,4; *F* = 9.049; *p* = 0.026), as well as in the second (d.f. = 6,4; *F* = 7.678; *p* = 0.034). Therefore the water layer in Bt rice fields harbored more abundant and diverse zooplankton than that of non-Bt rice.

**Table 1:** Abundance (individuals L^−1^) and diversity (number of species) of Rotifera, Cladocera and Copepoda in Bt and non-Bt rice plots.

Treatment	Rotifera	Cladocera	Copepoda	Total
^1^st investigation (abundance)				
non-Bt rice	10.33±5.17b	0±0b	35.33±25.56b	45.66±29.55b
Bt rice 1	172.66±74.99a	29.00±7.57a	1501.00±651.00a	1702.66±688.26a
Bt rice 2	254.67±119.32a	56.26±12.33a	1031.67±335.56a	1342.50±348.50a
^1^st investigation (number of species)				
non-Bt rice	1.33±0.67a	0±0b	1.33±0.67b	2.67±1.33b
Bt rice 1	4.67±1.20a	7.00±1.00a	3.67±0.33a	15.33±1.20a
Bt rice 2	3.67±0.33a	6.33±2.08a	3.63±0.33a	15.70±3.00a
^2^nd investigation (abundance)				
non-Bt rice	425.33±107.33b	4.66±2.33b	148.00±43.39b	638.00±69.51b
Bt rice 1	1291.33±201.17a	34.33±8.76a	598.00±150.89a	1923.67±325.42a
Bt rice 2	829.67±25.69a	38.33±10.84a	521.33±50.11a	1317.33±63.89a
^2^nd investigation (number of species)				
non-Bt rice	4.33±0.67a	2.00±0.58b	3.00±0.58a	9.33±0.88b
Bt rice 1	5.67±0.33a	7.00±1.53a	3.67±0.33a	16.33±1.86a
Bt rice 2	5.00±0.00a	6.00±0.00a	4.00±0.00a	15.00±0.00a

Non-Bt and Bt rice plots were sprayed with pesticides when rice pests exceeded the action threshold (Table S1). The first and the second investigation were conducted 5 days and 12 days after the final spray, respectively. The values presented in the table are untransformed means ± SE of each treatment. Means followed by the same letter within the same column in each investigation do not differ significantly at *p* = 0.05 (LSD post hoc test after ANOVA, n = 3).

### Laboratory rearing of *D. magna* and *P. caudatum*

**Figure 2:**
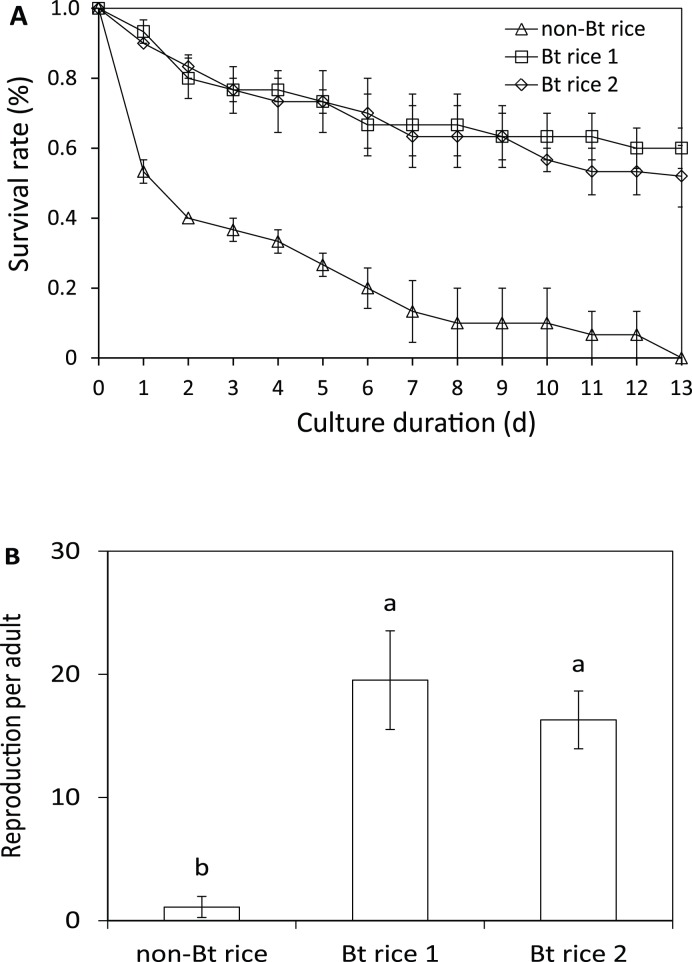
Survival (A) and reproductive rate (B) of *D. magna* reared in water collected from non-Bt rice and Bt rice plots. Water was collected 6 days after the final pesticide sprays and the culture medium was renewed every 3 days with field water collected on that very day. Non-Bt and Bt rice plots were sprayed with pesticides when rice pests exceeded the action threshold (see Table S1). Error bars indicated standard error. Different letters capped on the bars indicate significant difference at *p* = 0.05 (LSD post hoc test after ANOVA).

We tested the survival of *D. magna* and *P. caudatum* in water collected from Bt and non-Bt rice plots in the laboratory. Daphnids (<24 hrs old) were reared in 100 ml field-collected water with *Scenedesmus obliquus* as feed, and every 3 days the culture medium was refreshed with field-collected water. Differences on survival rate were found between Bt and non-Bt field-collected water one day after the onset of this experiment (Fig. 2A). In following days, survival rate in the non-Bt treatment decreased gradually to zero by the end of this 13-day experiment, while survival rates in the two Bt treatments remained relatively stable at 52% and 60% by day 13. Repeated-measures ANOVA within RCBD found significant rice type effect (d.f. = 2,4; *F* = 34.393, *p* = 0.003) and measuring time effect (d.f. = 12,52; *F* = 59.489; *p*<0.001), and no significant block effect (d.f. = 2,4; *F* = 1.587; *p* = 0.311). The survival rate did not differ between the two Bt rice treatments (LSD test, *p* = 0.549), but did between non-Bt rice and Bt rice 1 or Bt rice 2 (LSD test, *p* = 0.002 and *p* = 0.003). Reproduction was observed in the culture medium on the 7th day of the experiment in all treatments, which indicated rice type had no effect on sexual maturity age of *D. magna*. A significant effect of rice type on average reproduction rate was observed (d.f. = 2,4; *F* = 8.963; *p* = 0.033), and LSD tests revealed significant differences between non-Bt rice and Bt rice 1 (*p* = 0.018) or Bt rice 2 (*p* = 0.026) (Fig. 2B). The growth (body length) of *D. magna* was also recorded, but no significant differences were found.

**Figure 3:**
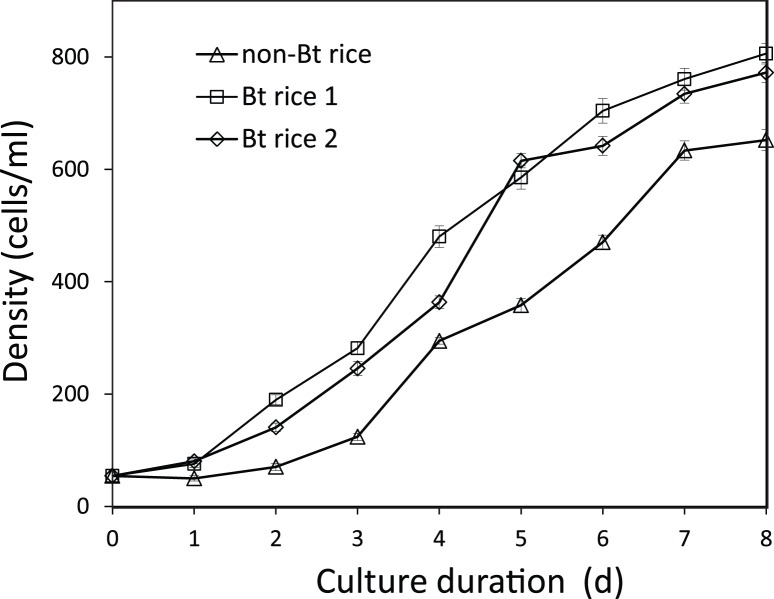
Population growth of *P. caudatum* in culture medium made of field water. Field water was collected from non-Bt rice and Bt rice plots 11 days after the final pesticide spray (see Table S1). Error bars indicate the standard error.

A stock of *P. caudatum* was cultured in the laboratory by traditional methods [29]. The stock culture was diluted to 54.3 cells ml^−1^ in 50 ml culture medium with 10 ml rice straw infusion and approx. 37 ml filtered field water collected from Bt and non-Bt plots. The population growth was inspected daily until the 8^th^ day. The *P. caudatum* expressed lower growth rate in non-Bt rice compared to the two Bt rice treatments (Fig. 3). Repeated-measures ANOVA within RCBD detected significant effects of rice type (d.f. = 2,4; *F* = 195.707; *p*<0.001), measuring time (d.f. = 8,32; *F* = 1790.116; *p*<0.001) and the interaction of rice type×measuring time (d.f. = 16,32; *F* = 13.109; *p*<0.001). The growth rates did not differ between Bt rice 1 and Bt rice 2 (LSD test, *p* = 0.072). The mean density was about 300 cells/ml in non- Bt culture, and 450 and 500 in Bt rice 1 and Bt rice 2 cultures by the end of this experiment.

### Pesticide residue in field-collected water

In our previous study, we revealed that Bt rice (with gene *cry1Ab/1Ac*) in itself has no negative impact on paddy zooplanktons [30]. In order to determine what caused the differences in survival found earlier, we tested pesticide residues in the water collected from the field 10 days after the final spray (see Table S1 for pesticide application dates). We found that residues of pesticides targeting Homopteran and fungal pests were in the same order of magnitude in Bt and non-Bt rice plots, since those pesticides were used both in Bt rice and non-Bt rice (Table 2). The residues of chlorantraniliprole and triazophos, targeting lepidopteran pests, were100 times higher in non-Bt plots than Bt plots (Table 2). Theoretically, pesticides targeting lepidopteran pests would not be detected in Bt plots because they were not used in Bt rice. However, they were detected in Bt plots probably because pesticide spray drifted by wind or/and water although we had made efforts to avoid this. In short, the pesticide residues in non-Bt plots were far higher than Bt plots, which likely accounts for the poor fitness performance of zooplanktons in the non-Bt treatment.

**Table 2:** Pesticide residue ± SE (µg L^−1^) in water collected from non-Bt and Bt rice plots.

Pesticide	Non-Bt rice	Bt rice 1	Bt rice 2
chlorantraniliprole	15.19±1.41	0.17±0.04	0.17±0.04
difenoconazole	5.44±1.37	1.47±0.89	2.57±1.38
propiconazole	35.21±1.15	42.67±14.92	26.93±8.89
triazophos	80.45±11.91	0.78±0.23	1.19±0.52
buprofezin	0.73±0.29	0.16±0.08	0.28±0.21

Field water was collected 10 days after the final spray (see table S1 for the spraying date). Pesticide residues were quantified by liquid chromatography-tandem mass spectrometry (LC-MS/MS).

## Discussion

Research on genetic modification has developed rapidly in China. In November 2009, COMA announced that biosafety licenses were issued to two lepidopteran-resistant Bt rice lines in designated areas. However, the decision was the subject of heated scientific and public debates in the following years. The core of these debates is the uncertainty regarding food safety and potential environmental risks.

In this study, we evaluated the environmental risk of two Bt rice lines from a new angle. Firstly, we focused on paddy aquatic organisms as the non-target organisms in the environmental assessment of Bt rice. Previous non-target effect studies of Bt crops mainly focuses on terrestrial invertebrates, such as earthworms, collembolans, mites, woodlice and nematodes [31]–[35]. Like those terrestrial invertebrates, aquatic organisms also carry out important ecological functions, such as nutrient cycling, decomposition of organic matter and maintaining aquatic food webs [36]. In many areas, the water matrix of rice fields is connected to nearby ponds and lakes which are important resources for rice farming farmers for additional income and food by fish production [37]. Secondly, we assessed Bt rice’s environmental effects in comparison with non-Bt rice under realistic agricultural conditions in which pesticides were used in both Bt and non-Bt fields as required. Former field studies demonstrated that although Bt rice (*cry1Ab/Ac*) reduced pesticide use by up to 60%, they were still required against fungal and non-lepidopteran pests in order to guarantee no yield loss [14], [15]. However, almost all previous studies on Bt rice’s non-target effect were conducted without considering the factor of pesticide application [4].

In this study, Bt rice was sprayed twice and non-Bt rice was sprayed 5 times throughout the whole growing season (Table S1), which resulted in equal yield between Bt and non-Bt rice (Fig. 1). The Bt rice expressed good resistance to lepidopteran pests, more specifically rice leaf folder and rice stem borers, and the two sprays only targeted a fungal disease and rice planthopper. The same results were reported in Bt cotton and Bt maize which also greatly reduced insecticide applications [38].

Our first field investigation found that the total abundance of three groups of zooplankton Rotifera, Cladocera and Copepoda in non-Bt field was about 95% lower than that in Bt rice field, and the total number of species was 80% lower (Table 1). The second sampling was conducted 7 days after the first one, and a rise in abundance and diversity was observed, however zooplanktons were still significantly more abundant and diverse in the Bt rice field. In contrast to this result, our previous two-year field study, in which we did not use pesticides, found the total population density of rotifera, cladocera and copepods did not differ significantly between Bt (*cry1Ab/1Ac*) and non-Bt rice fields [30]. The differences we report here are therefore not due to Bt rice itself but from the differences in pesticide application. We tested pesticide residues using LC-MS/MS in water samples collected from rice fields and found that pesticide (targeting lepidopteran pests) residues were around 100 times higher in non-Bt than Bt plots (Table 2). It is widely documented that pesticides enter into aquatic environments and accumulate to impact certain aquatic organisms. Some zooplanktons are frequently used in ecotoxicological tests of anthropogenic chemicals [39]. The qualitative and quantitative characteristics of zooplankton population depend on the degree of water pollution [40]. The high abundance of zooplankton in Bt rice fields corresponds with low water pollution from reduced pesticide application, and can be considered a positive effect of Bt rice on aquatic environment.

In addition to field investigation, we conducted laboratory experiment to rear *D. magna* and *P. caudatum* in field-collected water. The survival rate of *D. magna* declined to zero in non-Bt rice field-collected water by the end of the 13-days rearing experiment, while the survival rate was up to 60% in Bt rice field water (Fig. 2A). The amount of reproduction in Bt rice field water was more than 15 times higher than in non-Bt rice field water (Fig. 2B). The population of *P. caudatum* also developed faster in Bt rice field-collected water, and the final population density was about 20% higher in Bt rice field-collected water than non-Bt rice water (Fig. 3). Up to now, only a few studies have assessed non-target effects of Bt crops on aquatic organisms. Rosi-Marshall and colleagues reported that consumption of Bt corn (*cry1Ab*) debris reduced growth and increased mortality of caddisfly larvae in laboratory feeding trials [41]. Bøhn’s study demonstrated that *D. magna* fed on Bt maize (*cry1Ab*) showed significantly reduced fitness performance compared to the non-Bt control [42]. Rosi-Marshall’s study concluded that Bt δ-endotoxin in Bt maize byproducts harmed aquatic trichopterans insects which are phylogenetically close to Lepidoptera, and Bøhn’s study concluded a toxic effect rather than a lower nutritional value of Bt maize accounting for the lower fitness of *D. magna*. Their findings were quite different from the results of our present study. Although Bt protein is sometimes detected in Bt rice field water [43], we did not consider Bt proteins to have negative impact on paddy *Daphnia* because a another water flea species, *Daphnia hyalina*, was shown to perform as well in culture medium with high concentrations of Bt toxin as in the control medium [30]. In this study, we cultured *D. magna* and *P. caudatum* just in water collected from rice field and did not feed with Bt rice materials because water flea and paramecium typically do not feed directly on rice products or by-products [44]. In their studies, the non-target organisms were fed with high doses of Bt maize materials for a longer time than ours. Nevertheless, those aquatic organisms would not use a single food source for that long in the natural environment. Furthermore, they did not consider agricultural chemicals (such as pesticides) that probably existed in their materials, making their conclusions questionable [45]. We found that field-collected water from Bt rice fields was more suitable for survive survival and reproduction of paddy zooplanktons in short-term rearing trials, and we also detected higher pesticide residues in the water from non-Bt rice fields than Bt rice fields. Our results therefore indicate that pesticide residues in field-collected water play a more important role in zooplankton fitness than Bt toxins and Bt rice, which highlight the importance of considering pesticide application in non-target environmental assessments of Bt crops.

## Conclusions

In order to settle the debate on Bt rice and realize its potential benefit as early as possible [46], scientific experiments are needed to reveal the real environmental effects of Bt rice. We assessed the influence of two Bt rice lines on aquatic organisms under normal field management which used pesticide when pests exceeded the action threshold. The results indicated that Bt rice required reduced pesticide application therefore produced lower pesticide residues in the field water body. The abundance and diversity of zooplankton were significantly higher in Bt rice fields than non-Bt rice fields, and *D. magna* and *P. caudatum* displayed far higher survival and fecundity in Bt rice field water than non-Bt rice field water. These results demonstrate that the Bt rice lines are more friendly to the aquatic ecosystem than conventional rice. Pesticide application in rice fields has long been blamed for biodiversity loss in rice*-*based ecosystems [47] and it is expected that biodiversity will gradually be restored following the release of Bt rice.

## Supporting Information

Table S1 **Details of pesticide application in non-Bt and Bt rice plots.**
(DOCX)

Table S2 **Zooplankton taxa found in non-Bt and Bt rice fields.**
(DOCX)
