# Supplementary material for: Transgenic Bacillus thuringiensis (Bt) Rice Is Safer to Aquatic Ecosystems than Its Non-Transgenic Counterpart
Source: PLoS One. 2014 Aug 8;9(8):e104270. doi: 10.1371/journal.pone.0104270 (PMC4126711; doi:10.1371/journal.pone.0104270)
Supplement: Table S1 — Details of pesticide application in non-Bt and Bt rice plots. (DOCX) [file pone.0104270.s001.docx]

Table S1 Details of pesticide application in non-Bt and Bt rice plots

| Date | Pesticide | Dosage | Target Pest | Plot type |
| --- | --- | --- | --- | --- |
| June 26 | avermectins | 18 g a.i. ha^-1^ | rice leaffolder | non-Bt |
| July 8 | chlorantraniliprole | 10 g a.i. ha^-1^ | rice stem borers | non-Bt |
| July 23 | triazophos | 132 g a.i. ha^-1^ | rice leaffolder & rice stem borers | non-Bt |
| July 29 | difenoconazole & propiconazole * | 27 g a.i. ha^-1^ | rice sheath blight disease | non-Bt and Bt |
| August 1 | buprofezin | 22.5 g a.i. ha^-1^ | rice planthoppers | non-Bt and Bt |

* 1:1 w**/**w mixture
