# Supplementary material for: Transgenic Bacillus thuringiensis (Bt) Rice Is Safer to Aquatic Ecosystems than Its Non-Transgenic Counterpart
Source: PLoS One. 2014 Aug 8;9(8):e104270. doi: 10.1371/journal.pone.0104270 (PMC4126711; doi:10.1371/journal.pone.0104270)
Supplement: Table S2 — Zooplankton taxa found in non-Bt and Bt rice fields. (DOCX) [file pone.0104270.s002.docx]

Table S2. Zooplankton taxa found in non-Bt and Bt rice fields

| Taxa | First Investigation | | | | Second Investigation | | |
| --- | --- | --- | --- | --- | --- | --- | --- |
|  | Non-Bt | Bt 1 | Bt 2 |  | Non-Bt | Bt 1 | Bt 2 |
| Rotifera |  |  |  | |  |  |  |
| *Brachionus capsuliflorus* (Pallas) | - | - | - | | - | + | - |
| *Brachionus quadridentatus (*Hermann) | - | + | - | | - | - | - |
| *Euchlanis dilatata (*Ehrenberg) | - | - | - | | + | - | - |
| *Lecane luna* (O. F. Müller) | - | + | + | | - | - | - |
| *Lecane papuana* (Murray) | - | + | + | | - | + | + |
| *Lecane* sp | - | - | - | | - | + | - |
| *Lepadella patella* (O. F. Müller) | - | + | - | | - | + | + |
| *Monostyla hamata* (Stokes) | - | - | - | | + | + | + |
| *Monostyla lunaris* (Ehrenberg) | - | + | + | | - | + | - |
| *Mytilina ventralis* (Ehrenberg) | - | - | - | | - | + | - |
| *Plalyias militaris* (Daday) | ++ | ++++ | +++ | | ++ | +++ | +++ |
| *Platyias quadricornis* (Ehrenberg) | + | - | - | | + | - | - |
| *Polyarthra* sp | - | + | - | | + | + | ++ |
| *Rotaria neptunia (*Ehrenberg) | - | - | - | | + | ++ | + |
| *Scaridium* sp | + | - | - | | - | - | - |
| *Sinantherina semibullata* (Thorpe) | - | +++ | ++++ | | +++ | ++++ | ++++ |
| *Testudinella caeca (*Parsons) | - | + | - | | - | - | - |
| Cladocera |  |  |  | |  |  |  |
| *Alona guttata* (Sars) | - | ++ | ++ | | + | ++ | + |
| *Alona quadrangularis* (O.F. Müller) | - | + | ++ | | + | + | + |
| Ceriodaphnia *cornuta* (Sars) | - | ++ | ++ | | + | ++ | + |
| *Daphnia cucullata* (Sars) | - | - | + | | - | - | - |
| *Diaphanosoma* sp | - | - | - | | + | ++ | ++ |
| *Euryalona orientalis* (Daday) | - | + | + | | - | - | - |
| *Kurzia latissima* (Kurz) | - | - | - | | + | + | + |
| *Leydigia* sp | - | + | - | | - | + | - |
| *Moina* sp | - | + | + | | + | + | ++ |
| *pleuroxus assimills* (Brady) | - | + | ++ | | - | + | - |
| *Pleuroxus laevis* (Sars) | - | + | + | | - | + | ++ |
| *Pleuroxus trigonellus* (O. F. Müller） | - | + | + | | - | ++ | ++ |
| *Scapholeberis kingi* (Sars) | - | + | + | | - | - | - |
| *Simocephalus vetulus* (O. F. Müller） | - | + | + | | - | + | + |
| Copepoda |  |  |  | |  |  |  |
| *Mesocylops* sp | + | + | ++ | | + | + | ++ |
| *Thermocyclops* sp | - | + | ++ | | + | + | + |

Bt 1 and Bt 2 refer to Bt rice 1 and Bt rice 2, respectively; -, not found; +, 0‒5 individuals L^-1^; ++, 5‒20 individuals L^-1^; +++, 21‒100 individuals L^-1^; ++++, >100 individuals L^-1^.
